# The genome sequence of the Small Square-spot, *Diarsia rubi* (Vieweg, 1790)

**DOI:** 10.12688/wellcomeopenres.19299.1

**Published:** 2023-05-10

**Authors:** Douglas Boyes, Peter W.H. Holland, University of Oxford and Wytham Woods Genome Acquisition Lab, Darwin Tree of Life Barcoding collective, Wellcome Sanger Institute Tree of Life programme, Wellcome Sanger Institute Scientific Operations: DNA Pipelines collective, Tree of Life Core Informatics collective, Darwin Tree of Life Consortium

**Affiliations:** 1 UK Centre for Ecology & Hydrology, Wallingford, England, UK; 2 University of Oxford, Oxford, England, UK

**Keywords:** Diarsia rubi, Small square-spot, genome sequence, chromosomal, Lepidoptera

## Abstract

We present a genome assembly from an individual female *Diarsia rubi* (the Small Square-spot; Arthropoda; Insecta; Lepidoptera; Noctuidae). The genome sequence is 624.9 megabases in span. Most of the assembly is scaffolded into 32 chromosomal pseudomolecules, including the Z and W sex chromosomes. The mitochondrial genome has also been assembled and is 15.3 kilobases in length. Gene annotation of this assembly on Ensembl identified 19,173 protein coding genes.

## Species taxonomy

Eukaryota; Metazoa; Ecdysozoa; Arthropoda; Hexapoda; Insecta; Pterygota; Neoptera; Endopterygota; Lepidoptera; Glossata; Ditrysia; Noctuoidea; Noctuidae; Noctuinae; Noctuini; *Diarsia*; *Diarsia rubi* (Vieweg, 1790) (NCBI:txid987925).

## Background

The genus *Diarsia* contains around 100 species of noctuid moths, found primarily across the Palaearctic, Indomalayan and Australasian realms; many *Diarsia* species have similar wing markings ( Gyulai & Saldaitis, 2019). Even within a region with low diversity in the genus, such as Britain and Ireland with just four or five species, it can sometimes be difficult to distinguish species using external characters alone. The Small Square-spot *D. rubi* is a common and often abundant moth found across England, Wales, Scotland, Northern Ireland and Ireland ( Randle *et al.*, 2019). The species is also common across most of Europe, with sporadic records from further east to Russia and Tajikistan ( GBIF Secretariat, 2022). The moth is frequent in gardens, woodlands and grassland habitats with the polyphagous larvae feeding on a wide range of herbaceous plants ( Skinner & Wilson, 2009; South, 1961).

The common name derives a square-shaped russet brown patch between the pale orbicular and reniform stigmata (oval and kidney marks), on a grey-brown ground colour. The same pattern is also present in *D. mendica*, which may be distinguished from *D. rubi* by subtle wing pattern differences and genitalia morphology ( Townsend *et al.,* 2010). *D. florida* lacks clear genitalic differences from *D. rubi*, and further work is needed to clarify if these are distinct species ( Skinner & Wilson, 2009; Townsend *et al.,* 2010).

The life cycle of *D. rubi* reveals an interesting case of environmental influence on development. Although the moth is single brooded in the north of its range including Scotland, further south across England and Wales the species has two generations each year that differ in appearance. Moths from the spring generation, on the wing in May and June, are larger and generally paler than moths from the summer generation, on the wing in August and September ( Skinner & Wilson, 2009; South, 1961). It is unclear if this is a consequence of diet, since the larvae giving rise to the spring emergence feed for longer ( Ford, 1967), or an environmental polymorphism triggered by a variable such as day length, analogous to the comma butterfly *Nymphalis c-album* see ( Nylin, 1989).

A complete genome sequence from *D. rubi* will facilitate research into adaptations to polyphagy, and the interaction between genes and environment.

### Genome sequence report

**Figure 1.**
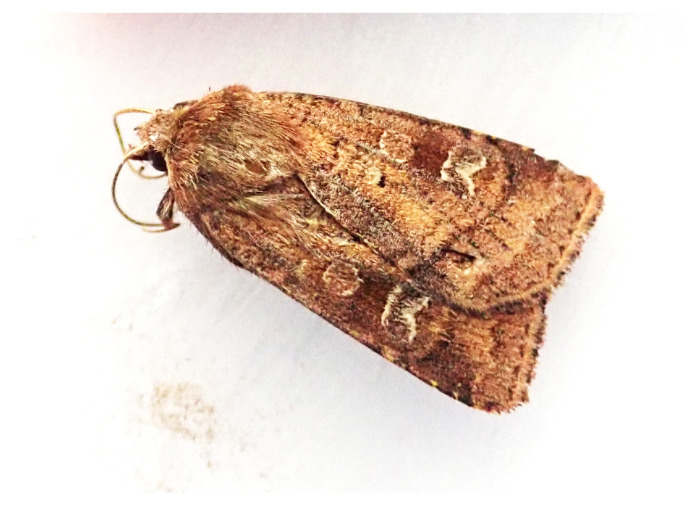
Photograph of the *Diarsia rubi* (ilDiaRubi1) specimen used for genome sequencing.

The genome was sequenced from one female *Diarsia rubi* specimen ( Figure 1) collected from Wytham Woods, Oxfordshire, UK (latitude 51.77, longitude –1.34). A total of 33-fold coverage in Pacific Biosciences single-molecule HiFi long reads and 52-fold coverage in 10X Genomics read clouds was generated. Primary assembly contigs were scaffolded with chromosome conformation Hi-C data. Manual assembly curation corrected 10 missing joins or mis-joins, reducing the scaffold number by 22.73%.

**Figure 2.**
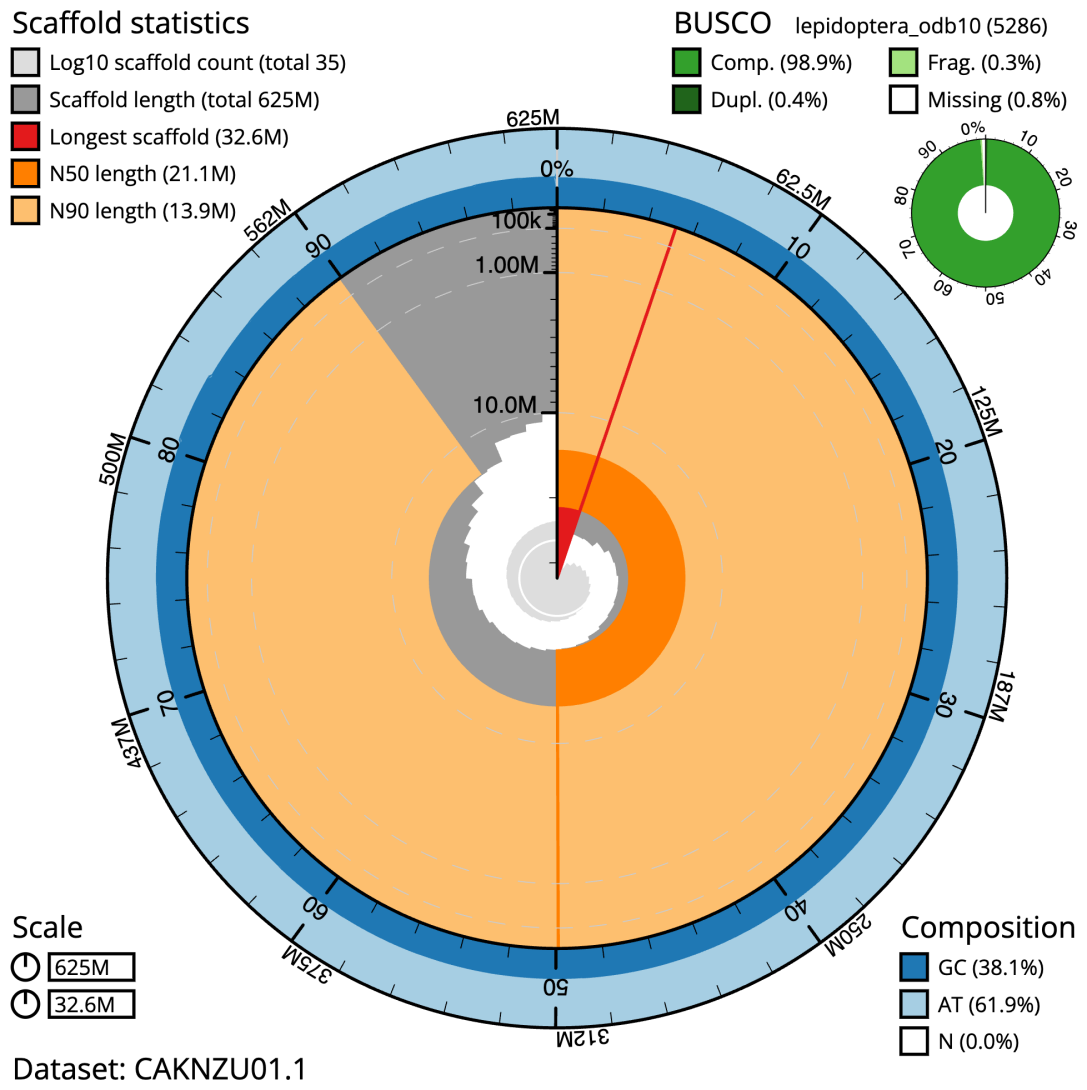
Genome assembly of *Diarsia rubi*, ilDiaRubi1.1: metrics. The BlobToolKit Snailplot shows N50 metrics and BUSCO gene completeness. The main plot is divided into 1,000 size-ordered bins around the circumference with each bin representing 0.1% of the 624,929,271 bp assembly. The distribution of scaffold lengths is shown in dark grey with the plot radius scaled to the longest scaffold present in the assembly (32,634,975 bp, shown in red). Orange and pale-orange arcs show the N50 and N90 scaffold lengths (21,075,383 and 13,933,945 bp), respectively. The pale grey spiral shows the cumulative scaffold count on a log scale with white scale lines showing successive orders of magnitude. The blue and pale-blue area around the outside of the plot shows the distribution of GC, AT and N percentages in the same bins as the inner plot. A summary of complete, fragmented, duplicated and missing BUSCO genes in the lepidoptera_odb10 set is shown in the top right. An interactive version of this figure is available at https://blobtoolkit.genomehubs.org/view/ilDiaRubi1.1/dataset/CAKNZU01.1/snail.

The final assembly has a total length of 624.9 Mb in 34 sequence scaffolds with a scaffold N50 of 21.1 Mb ( Table 1). Most (99.99%) of the assembly sequence was assigned to 32 chromosomal-level scaffolds, representing 30 autosomes, and the Z and W sex chromosomes. Chromosome-scale scaffolds confirmed by the Hi-C data are named in order of size ( Figure 2– Figure 5; Table 2). While not fully phased, the assembly deposited is of one haplotype. Contigs corresponding to the second haplotype have also been deposited. The mitochondrial genome was also assembled and can be found as a contig within the multifasta file of the genome submission.

**Table 1.** Genome data for *Diarsia rubi*, ilDiaRubi1.1.

Project accession data		
Assembly identifier	ilDiaRubi1.1	
Species	*Diarsia rubi*	
Specimen	ilDiaRubi1	
NCBI taxonomy ID	987925	
BioProject	PRJEB50480	
BioSample ID	SAMEA8603186	
Isolate information	ilDiaRubi1, female: thorax (genome sequencing), head (Hi-C scaffolding)	
Assembly metrics *		*Benchmark*
Consensus quality (QV)	61.2	*≥ 50*
*k*-mer completeness	100%	*≥ 95%*
BUSCO **	C:98.9%[S:98.4%,D:0.4%], F:0.3%,M:0.8%,n:5,286	*C ≥ 95%*
Percentage of assembly mapped to chromosomes	99.99%	*≥ 95%*
Sex chromosomes	Z and W chromosomes	*localised homologous pairs*
Organelles	Mitochondrial genome assembled	*complete single alleles*
Raw data accessions		
PacificBiosciences SEQUEL II	ERR8482055, ERR8482056	
10X Genomics Illumina	ERR8373776–ERR8373779	
Hi-C Illumina	ERR8373775	
Genome assembly		
Assembly accession	GCA_932274075.1	
*Accession of alternate haplotype*	GCA_932274355.1	
Span (Mb)	624.9	
Number of contigs	46	
Contig N50 length (Mb)	21.1	
Number of scaffolds	34	
Scaffold N50 length (Mb)	21.1	
Longest scaffold (Mb)	32.6	
Genome annotation		
Number of protein-coding genes	19,173	
Number of gene transcripts	19,371	

* Assembly metric benchmarks are adapted from column VGP-2020 of “Table 1: Proposed standards and metrics for defining genome assembly quality” from ( Rhie *et al.*, 2021). ** BUSCO scores based on the lepidoptera_odb10 BUSCO set using v5.3.2. C = complete [S = single copy, D = duplicated], F = fragmented, M = missing, n = number of orthologues in comparison. A full set of BUSCO scores is available at https://blobtoolkit.genomehubs.org/view/ilDiaRubi1.1/dataset/CAKNZU01.1/busco.

**Figure 3.**
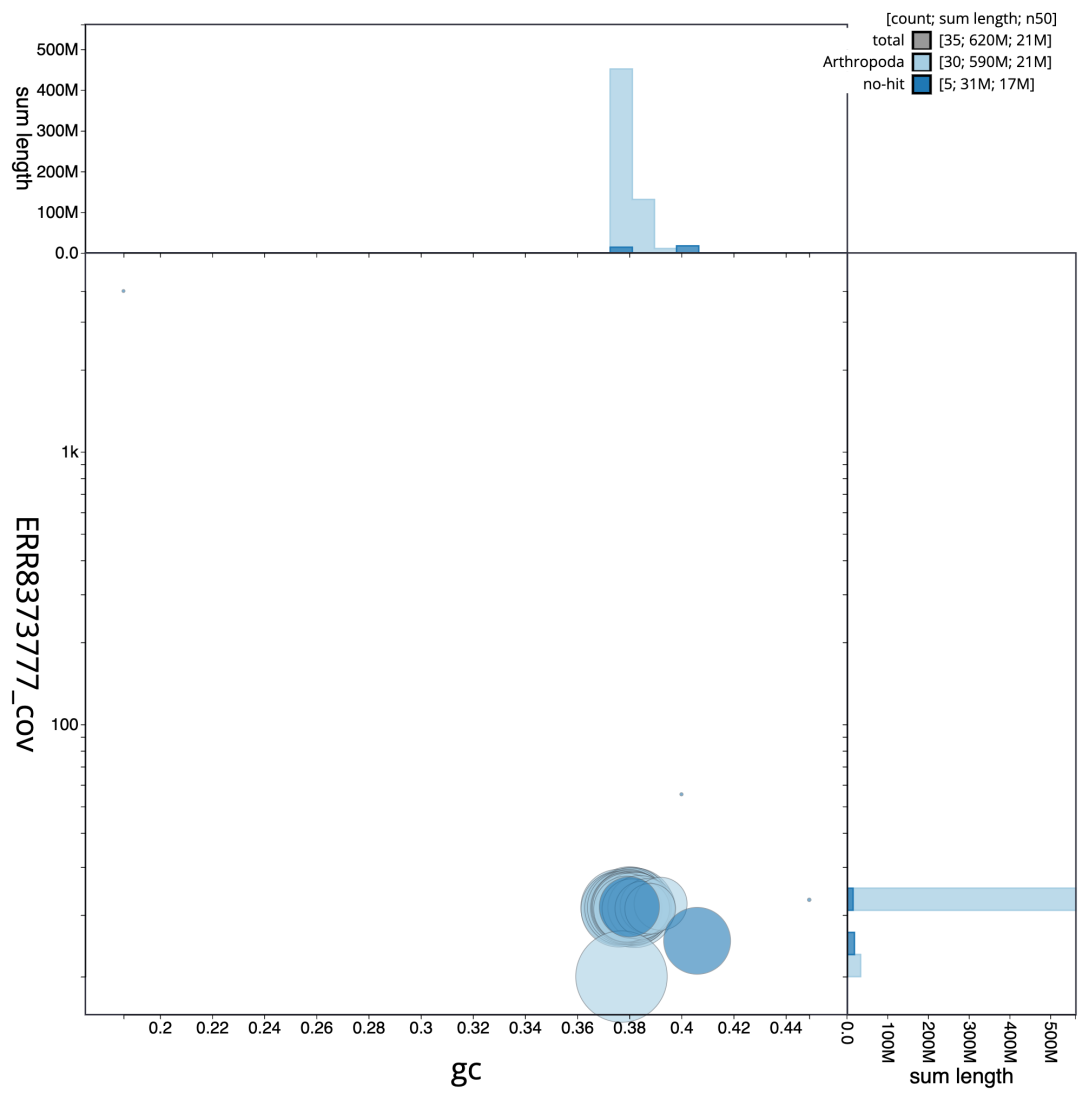
Genome assembly of *Diarsia rubi*, ilDiaRubi1.1: GC coverage. BlobToolKit GC-coverage plot. Scaffolds are coloured by phylum. Circles are sized in proportion to scaffold length. Histograms show the distribution of scaffold length sum along each axis. An interactive version of this figure is available at https://blobtoolkit.genomehubs.org/view/ilDiaRubi1.1/dataset/CAKNZU01.1/blob.

**Figure 4.**
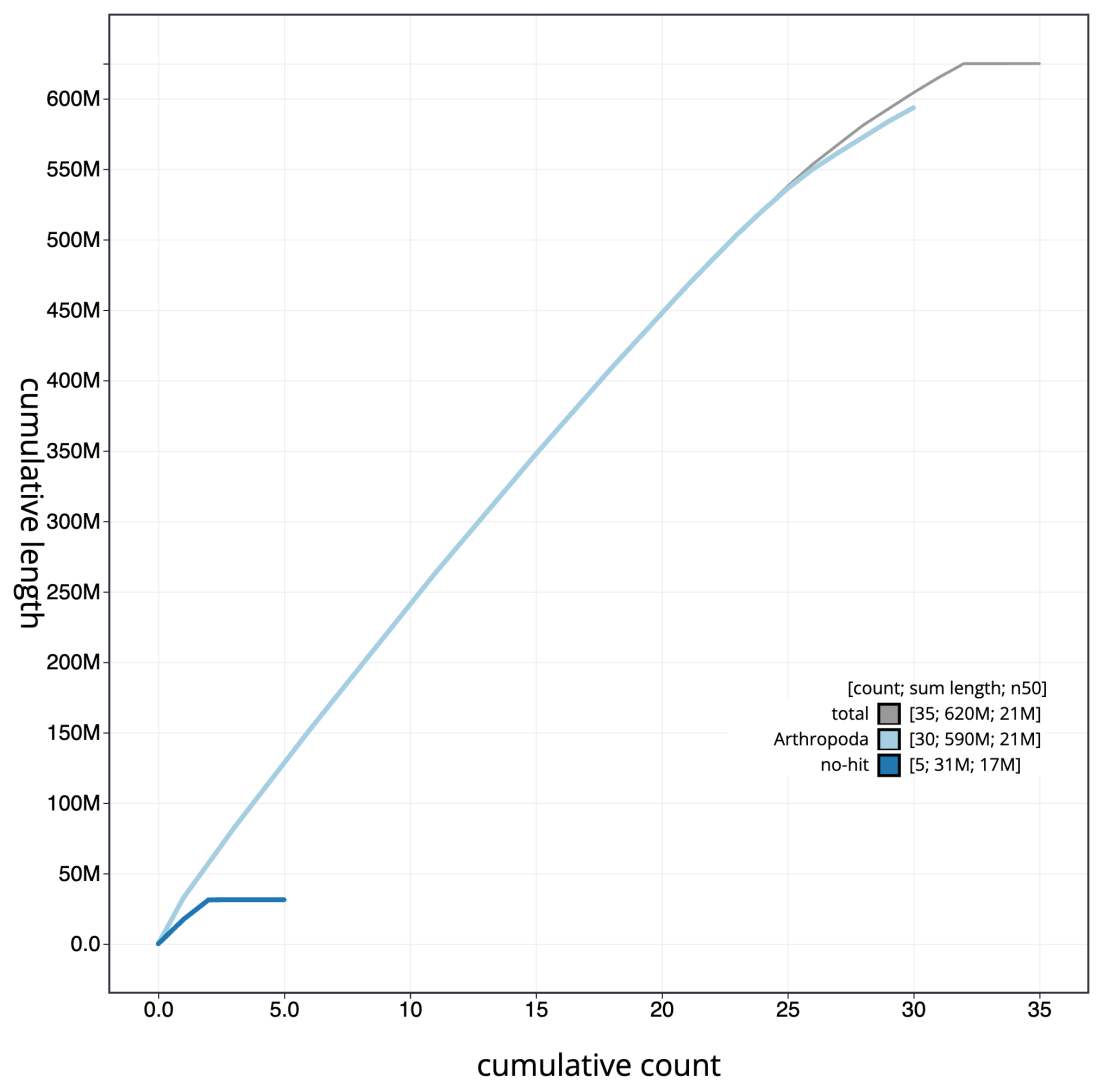
Genome assembly of *Diarsia rubi*, ilDiaRubi1.1: cumulative sequence. BlobToolKit cumulative sequence plot. The grey line shows cumulative length for all scaffolds. Coloured lines show cumulative lengths of scaffolds assigned to each phylum using the buscogenes taxrule. An interactive version of this figure is available at https://blobtoolkit.genomehubs.org/view/ilDiaRubi1.1/dataset/CAKNZU01.1/cumulative.

**Figure 5.**
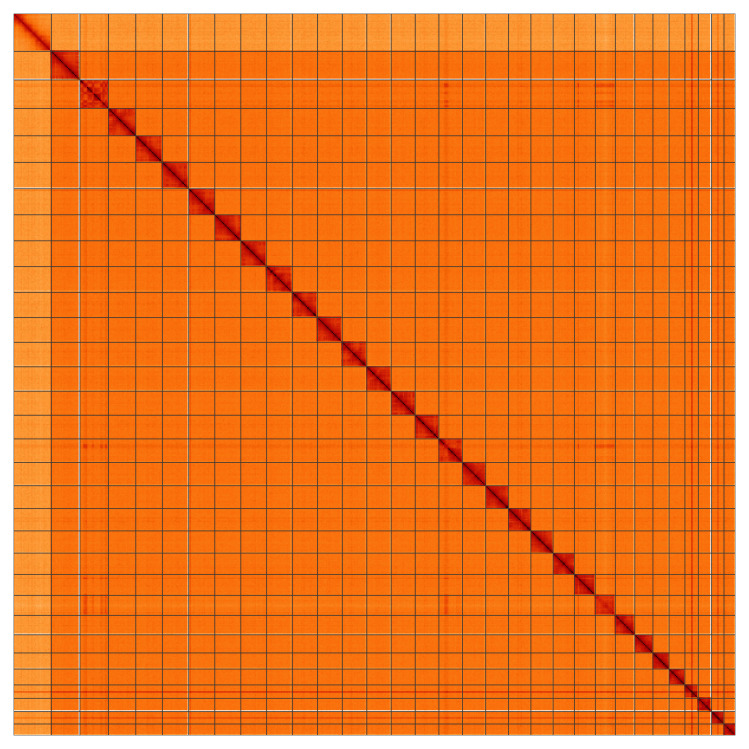
Genome assembly of *Diarsia rubi*, ilDiaRubi1.1: Hi-C contact map. Hi-C contact map of the ilDiaRubi1.1 assembly, visualised using HiGlass. Chromosomes are shown in order of size from left to right and top to bottom. An interactive version of this figure may be viewed at https://genome-note-higlass.tol.sanger.ac.uk/l/?d=RZTqYGymTpKj7IKceK8PxQ.

**Table 2.** Chromosomal pseudomolecules in the genome assembly of *Diarsia rubi*, ilDiaRubi1.

INSDC accession	Chromosome	Size (Mb)	GC%
OW026412.1	1	24.77	38
OW026413.1	2	24.63	38.2
OW026414.1	3	23.72	38.1
OW026415.1	4	22.99	38.1
OW026416.1	5	22.83	37.9
OW026417.1	6	22.59	37.7
OW026418.1	7	22.52	37.6
OW026419.1	8	22.37	37.8
OW026420.1	9	22.21	37.6
OW026421.1	10	21.88	38
OW026422.1	11	21.38	37.9
OW026423.1	12	21.24	38
OW026424.1	13	21.08	37.7
OW026425.1	14	20.83	37.8
OW026426.1	15	20.51	38
OW026427.1	16	20.32	38.2
OW026428.1	17	20.2	37.9
OW026429.1	18	19.78	38.1
OW026430.1	19	19.32	38.2
OW026431.1	20	19.22	37.9
OW026432.1	21	18.42	38.2
OW026433.1	22	18.17	37.9
OW026435.1	23	16.83	37.9
OW026436.1	24	15.71	38.4
OW026437.1	25	13.93	38
OW026438.1	26	13.75	38.1
OW026439.1	27	11.6	38.7
OW026440.1	28	11.48	38.5
OW026441.1	29	10.76	39.2
OW026442.1	30	9.85	38.8
OW026434.1	W	17.31	40.6
OW026411.1	Z	32.64	37.7
OW026443.1	MT	0.02	18.9

The estimated Quality Value (QV) of the final assembly is 61.2 with *k*-mer completeness of 100%, and the assembly has a BUSCO v5.3.2 completeness of 98.9% (single 98.4%, duplicated 0.4%) using the lepidoptera_odb10 reference set ( *n* = 5,286).

Metadata for specimens, spectral estimates, sequencing runs, contaminants and pre-curation assembly statistics can be found at https://links.tol.sanger.ac.uk/species/987925.

### Genome annotation report

The GCA_932274075.1 genome was annotated using the Ensembl rapid annotation pipeline ( Table 1; https://rapid.ensembl.org/Diarsia_rubi_GCA_932274075.1/Info/Index). The resulting annotation includes 19,371 transcribed mRNAs from 19,173 protein-coding genes.

## Methods

### Sample acquisition and nucleic acid extraction

A female *Diarsia rubi* (ilDiaRubi1) was collected from Wytham Woods, Oxfordshire (biological vice-county: Berkshire), UK (latitude 51.77, longitude –1.34) on 8 September 2020. The specimen was taken from woodland habitat by Douglas Boyes (University of Oxford) using a light trap. The specimen was identified by the collector and snap-frozen on dry ice.

DNA was extracted at the Tree of Life laboratory, Wellcome Sanger Institute (WSI). The ilDiaRubi1 sample was weighed and dissected on dry ice with head tissue set aside for Hi-C sequencing. Thorax tissue was cryogenically disrupted to a fine powder using a Covaris cryoPREP Automated Dry Pulveriser, receiving multiple impacts. High molecular weight (HMW) DNA was extracted using the Qiagen MagAttract HMW DNA extraction kit. Low molecular weight DNA was removed from a 20 ng aliquot of extracted DNA using the 0.8X AMpure XP purification kit prior to 10X Chromium sequencing; a minimum of 50 ng DNA was submitted for 10X sequencing. HMW DNA was sheared into an average fragment size of 12–20 kb in a Megaruptor 3 system with speed setting 30. Sheared DNA was purified by solid-phase reversible immobilisation using AMPure PB beads with a 1.8X ratio of beads to sample to remove the shorter fragments and concentrate the DNA sample. The concentration of the sheared and purified DNA was assessed using a Nanodrop spectrophotometer and Qubit Fluorometer and Qubit dsDNA High Sensitivity Assay kit. Fragment size distribution was evaluated by running the sample on the FemtoPulse system.

### Sequencing

Pacific Biosciences HiFi circular consensus and 10X Genomics read cloud DNA sequencing libraries were constructed according to the manufacturers’ instructions. DNA sequencing was performed by the Scientific Operations core at the WSI on Pacific Biosciences SEQUEL II (HiFi) and Illumina NovaSeq 6000 (10X) instruments. Hi-C data were also generated from head tissue of ilDiaRubi1 using the Arima v2 kit and sequenced on the Illumina NovaSeq 6000 instrument.

### Genome assembly, curation and evaluation

Assembly was carried out with Hifiasm ( Cheng *et al.*, 2021) and haplotypic duplication was identified and removed with purge_dups ( Guan *et al.*, 2020). One round of polishing was performed by aligning 10X Genomics read data to the assembly with Long Ranger ALIGN, calling variants with FreeBayes ( Garrison & Marth, 2012). The assembly was then scaffolded with Hi-C data ( Rao *et al.*, 2014) using YaHS ( Zhou *et al.*, 2023). The assembly was checked for as described previously ( Howe *et al.*, 2021). Manual curation was performed using gHiGlass ( Kerpedjiev *et al.*, 2018) and Pretext ( Harry, 2022). The mitochondrial genome was assembled using MitoHiFi ( Uliano-Silva *et al.*, 2022), which runs MitoFinder ( Allio *et al.*, 2020) or MITOS ( Bernt *et al.*, 2013) and uses these annotations to select the final mitochondrial contig and to ensure the general quality of the sequence. To evaluate the assembly, MerquryFK was used to estimate consensus quality (QV) scores and *k*-mer completeness ( Rhie *et al.*, 2020). The genome was analysed within the BlobToolKit environment ( Challis *et al.*, 2020) and BUSCO scores ( Manni *et al.*, 2021; Simão *et al.*, 2015) were calculated. Table 3 contains a list of software tool versions and sources.

**Table 3.** Software tools: versions and sources.

Software tool	Version	Source
BlobToolKit	4.0.7	https://github.com/blobtoolkit/blobtoolkit
BUSCO	5.3.2	https://gitlab.com/ezlab/busco
FreeBayes	1.3.1-17- gaa2ace8	https://github.com/freebayes/freebayes
Hifiasm	0.15.3	https://github.com/chhylp123/hifiasm
HiGlass	1.11.6	https://github.com/higlass/higlass
Long Ranger ALIGN	2.2.2	https://support.10xgenomics.com/genome-exome/software/pipelines/latest/advanced/ other-pipelines
Merqury	MerquryFK	https://github.com/thegenemyers/MERQURY.FK
MitoHiFi	2	https://github.com/marcelauliano/MitoHiFi
PretextView	0.2	https://github.com/wtsi-hpag/PretextView
purge_dups	1.2.3	https://github.com/dfguan/purge_dups
YaHS	1	https://github.com/c-zhou/yahs

### Genome annotation

The BRAKER2 pipeline ( Brůna *et al.*, 2021) was used in the default protein mode to generate annotation for the *Diarsia rubi* assembly (GCA_932274075.1) in Ensembl Rapid Release.

### Ethics and compliance issues

The materials that have contributed to this genome note have been supplied by a Darwin Tree of Life Partner. The submission of materials by a Darwin Tree of Life Partner is subject to the Darwin Tree of Life Project Sampling Code of Practice. By agreeing with and signing up to the Sampling Code of Practice, the Darwin Tree of Life Partner agrees they will meet the legal and ethical requirements and standards set out within this document in respect of all samples acquired for, and supplied to, the Darwin Tree of Life Project. All efforts are undertaken to minimise the suffering of animals used for sequencing. Each transfer of samples is further undertaken according to a Research Collaboration Agreement or Material Transfer Agreement entered into by the Darwin Tree of Life Partner, Genome Research Limited (operating as the Wellcome Sanger Institute), and in some circumstances other Darwin Tree of Life collaborators.

## Data Availability

European Nucleotide Archive: *Diarsia rubi* (small square-spot). Accession number PRJEB50480; https://identifiers.org/ena.embl/PRJEB50480. ( Wellcome Sanger Institute, 2022) The genome sequence is released openly for reuse. The *Diarsia rubi* genome sequencing initiative is part of the Darwin Tree of Life (DToL) project. All raw sequence data and the assembly have been deposited in INSDC databases. Raw data and assembly accession identifiers are reported in Table 1.

## Author information

Members of the University of Oxford and Wytham Woods Genome Acquisition Lab are listed here: https://doi.org/10.5281/zenodo.4789928.

Members of the Darwin Tree of Life Barcoding collective are listed here: https://doi.org/10.5281/zenodo.4893703.

Members of the Wellcome Sanger Institute Tree of Life programme are listed here: https://doi.org/10.5281/zenodo.4783585.

Members of Wellcome Sanger Institute Scientific Operations: DNA Pipelines collective are listed here: https://doi.org/10.5281/zenodo.4790455.

Members of the Tree of Life Core Informatics collective are listed here: https://doi.org/10.5281/zenodo.5013541.

Members of the Darwin Tree of Life Consortium are listed here: https://doi.org/10.5281/zenodo.4783558.
