# Supplementary material for: The genome sequence of the Small Square-spot, Diarsia rubi (Vieweg, 1790)
Source: Wellcome Open Res. 2023 May 10;8:210. [Version 1] doi: 10.12688/wellcomeopenres.19299.1 (PMC11358679; doi:10.12688/wellcomeopenres.19299.1)
Supplement: Peer review history

# Reviewer report: Reviewer response for version 1

**DOI:** 10.21956/wellcomeopenres.21382.r82358

**Published:** 2024-08-28

**Authors:** Huai-Jun Xue

**Affiliations:** 1 Nankai University, Tianjin, Tianjin, China

In the present study, based on HiFi circular consensus, 10X Genomics and HiC sequencing data, the authors present a high quality genome assembly of an individual female of the Small Square-spot, *Diarsia rubi.* The methods of sequencing, assembling and annotation are feasible, and the results look solid. I have no comments.

Are sufficient details of methods and materials provided to allow replication by others?

Yes

Is the rationale for creating the dataset(s) clearly described?

Yes

Are the datasets clearly presented in a useable and accessible format?

Yes

Are the protocols appropriate and is the work technically sound?

Yes

Reviewer Expertise:

Speciation

I confirm that I have read this submission and believe that I have an appropriate level of expertise to confirm that it is of an acceptable scientific standard.

# Reviewer report: Reviewer response for version 1

**DOI:** 10.21956/wellcomeopenres.21382.r77518

**Published:** 2024-08-28

**Authors:** Paula Escuer

**Affiliations:** 1 University of Neuchâtel, Neuchâtel, Switzerland

In this research article is presented the genome sequence of a female individual of *Diarsia rubi* (the Small Square-spot; Arthropoda; Insecta; Lepidoptera; Noctuidae). The assembly size contains 624.9 Mb condensed in 34 scaffolds with a scaffold N50 of 21.1 Mb (Table 1, Figure 5). The 99.9% of the assembly is distributed across 32 chromosomal level scaffolds, which represents the 30 autosomes plus the Z and W sex chromosomes. The mitochondrial genome is available and comprises 15.3 kb of length. The BUSCO pipeline identified 98.9% of complete genes, indicating a high completeness of the genome (Table 3, Figure 2). Finally, they identified 19,173 protein-coding genes using the default protein mode of the annotation pipeline BRAKER. This high quality genome will help to resolve questions about adaptations to their characteristic polyphagy and the evolution of Lepidoptera. 

 The article is clear and well written, they used standard protocols and pipelines for the sequencing and to assembly the genome. The resulting dataset presents good quality statistics, high contiguity and completeness, I recommend using RNA-seq data to improve the quality of annotation if possible.

Are sufficient details of methods and materials provided to allow replication by others?

Yes

Is the rationale for creating the dataset(s) clearly described?

Yes

Are the datasets clearly presented in a useable and accessible format?

Yes

Are the protocols appropriate and is the work technically sound?

Yes

Reviewer Expertise:

Biodiversity, Genomics, Speciation, Genome assembly and annotation

I confirm that I have read this submission and believe that I have an appropriate level of expertise to confirm that it is of an acceptable scientific standard.
